# The impact of *JAK2* ^V617F^ variant allele frequency in MPN patients following PEGylated interferon alpha discontinuation

**DOI:** 10.1111/bjh.70198

**Published:** 2025-10-24

**Authors:** Ryan Brown, Rebekah Bennett, Clare Crean, Andrew Hindley, Julie McGimpsey, Nicholas Cunningham, Aaron Niblock, Suzanne McPherson, Kathryn Boyd, Allister Foy, Ciaren Graham, Graeme Greenfield, Mary Frances McMullin, Mark A. Catherwood

**Affiliations:** ^1^ Regional Molecular Diagnostic Service Belfast Health and Social Care Trust Belfast Northern Ireland, UK; ^2^ School of Biological Sciences Queen's University Belfast Belfast Northern Ireland, UK; ^3^ Haematology Department Belfast Health and Social Care Trust Belfast Northern Ireland, UK; ^4^ Haematology Department Northern Health and Social Care Trust Antrim Northern Ireland, UK; ^5^ Haematology Department Southern Health and Social Care Trust Craigavon Northern Ireland, UK; ^6^ Centre for Medical Education Queens University Belfast Belfast Northern Ireland, UK

**Keywords:** *JAK2*, myeloproliferative neoplasm, pegylated interferon alpha

## Abstract

Treatment with pegylated interferon α resulted in a molecular response in 91% of patients with an average decrease in *JAK2* ^V61F^ VAF of 48.5% from baseline. In patients that discontinued treatment, the *JAK2* ^V617F^ VAF at the time of treatment discontinuation was the best indicator of durable remission over the 6‐month follow‐up period. Created with SMART Servier Medical Art (https://smart.servier.com/).

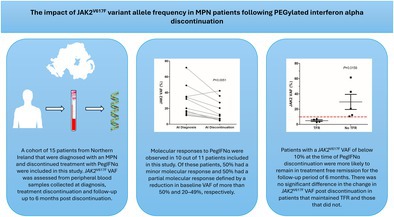

To the Editor,

Myeloproliferative neoplasms (MPN) are a group of conditions characterised by the clonal expansion of myeloid stem cells. MPNs are subclassified into three distinct entities, polycythaemia vera (PV), essential thrombocythaemia (ET) and primary myelofibrosis (PMF) based on the cell type in which the proliferation occurs and the presence/absence of bone marrow fibrosis.^1^ The most common driver in MPNs is a missense variant in the *JAK2* gene denoted as *JAK2* ^V617F^. The management of MPNs is primarily aimed at controlling cell counts and the associated risk of thrombosis.^2^ Therefore, cytoreductive therapy is recommended particularly in patients with high‐risk disease.^3^ Several cytoreductive therapies are available including the chemotherapy drug hydroxycarbamide, a JAK inhibitor ruxolitinib or recombinant interferon alpha (IFNα).^4^ IFNα is a cytokine whose primary physiological role is in anti‐viral immune responses. However, it has also been shown to mediate anti‐proliferative activity via the JAK1/STAT1 pathway.^5^ There are a number of pegylated IFNs (PegIFNα) in clinical use including peginterferon alpha‐2a (Pegasys) and ropeginterferon alpha‐2b‐njft (BESREMi). Use of BESREMi over Pegasys is increasing due to a similar safety profile and requirement for less frequent dosing.^6^ However, BESREMi is not currently reimbursed in Northern Ireland; as such, PegIFNα in this study refers to Pegasys. PegIFNα has emerged as a disease‐modifying agent in PV and ET, primarily due to its capacity to induce haematological and molecular responses and potentially eradicate the malignant clone.^7^, ^8^, ^9^ Treatment‐free remission (TFR), a concept long established in chronic myeloid leukaemia (CML), is now garnering attention in the field of Philadelphia‐negative MPNs, particularly in patients with *JAK2* ^V617F^‐positive disease. Importantly, previous studies have shown PegIFNα to provide long‐term complete haematological remission (CHR) in some patients following treatment discontinuation raising the prospect of TFR in these patients.^10^ Recent global supply chain issues of PegIFNα have meant that some MPN patients have been required to discontinue treatment with PegIFNα swapping to other approved treatments including hydroxycarbamide and ruxolitinib. As a result, this has provided an opportunity to study the possibility of achieving TFR in patients with a sustained haematological response.

The aim of this study was to assess the effect of PegIFN treatment on *JAK2* ^V617F^ variant allele frequency (VAF) and the impact on haematological parameters in a local cohort of MPN patients. Additionally, we aim to determine if *JAK2* ^V617F^ VAF correlates with TFR and may be an early predictor of haematological relapse.

A cohort of 15 patients diagnosed with an MPN according to the WHO 2022 diagnostic criteria and who discontinued treatment with PegIFNα between September and December 2024 were included in this study (Table S1). This cohort included eight PV patients and seven ET patients with a median age of 59 and 47.8% (7/15) male. Prior thrombotic events were recorded in 26.7% (4/15) of patients. A peripheral blood (PB) sample was collected at the point of treatment discontinuation and at 3‐ to 6‐month intervals post discontinuation. Following haematological relapse, as indicated by the return of MPN‐typical changes in the PB (erythrocytosis/thrombocytosis above the WHO 2022 diagnostic criteria) or clinical symptoms, treatment was reinitiated and an additional PB sample collected. Treatment choice was at the discretion of the treating clinician (Figure S1). Quantitative PCR (QPCR) was performed on DNA extracted from peripheral blood samples to determine *JAK2* ^V617F^ VAF. Further methodological details are included in the supplementary data.

Initially, we determined the effect of pegIFNα treatment on *JAK2* ^V617F^ VAF and clinical outcomes in a subset of patients for which we had a *JAK2* ^V617F^ VAF at both diagnosis and treatment discontinuation. A molecular response was observed in 91% (10/11) of patients treated with PegIFNα. None of the patients demonstrated a major molecular response (MMR) defined by a *JAK2* ^V617F^ < 2%. Of the patients who had a molecular response, 50% (5/10) had a minor molecular response and 50% (5/10) had a partial molecular response defined by a reduction in baseline VAF of more than 50% and 20%–49% respectively. Overall, a significant decrease in *JAK2* ^V617F^ VAF of 42.3% from baseline was observed in PegIFNα treated patients (Figure 1A). A mean rate of decrease in *JAK2* ^V617F^ VAF of 0.6% per month was observed across the study duration (Figure 1B). This is in line with larger trial data showing a monthly decline in *JAK2* ^V617F^ VAF of around 1%.^11^, ^12^ Of note, prior thrombotic events and MPN subclassification had no impact on molecular response to pegIFNα treatment (Figure S2A,B). These data are in keeping with several clinical trials in MPN patients which have demonstrated similar molecular responses to pegIFNα.^11^, ^13^ This finding has clinical relevance as *JAK2* ^V617F^ VAF correlates with more aggressive disease features and an increased risk of thrombotic events.^14^, ^15^ Additionally, *JAK2* ^V617F^ VAF reductions of >35% after 2 years of treatment have been associated with reduced progression to myelofibrosis.^16^ All clinical outcomes including haemoglobin concentration, haematocrit, platelet count and white cell count were significantly reduced by pegIFNα treatment (Figure 1C–F). Only one patient failed to maintain a haematological response while on pegIFNα treatment due to a loss of platelet response.

**FIGURE 1:**
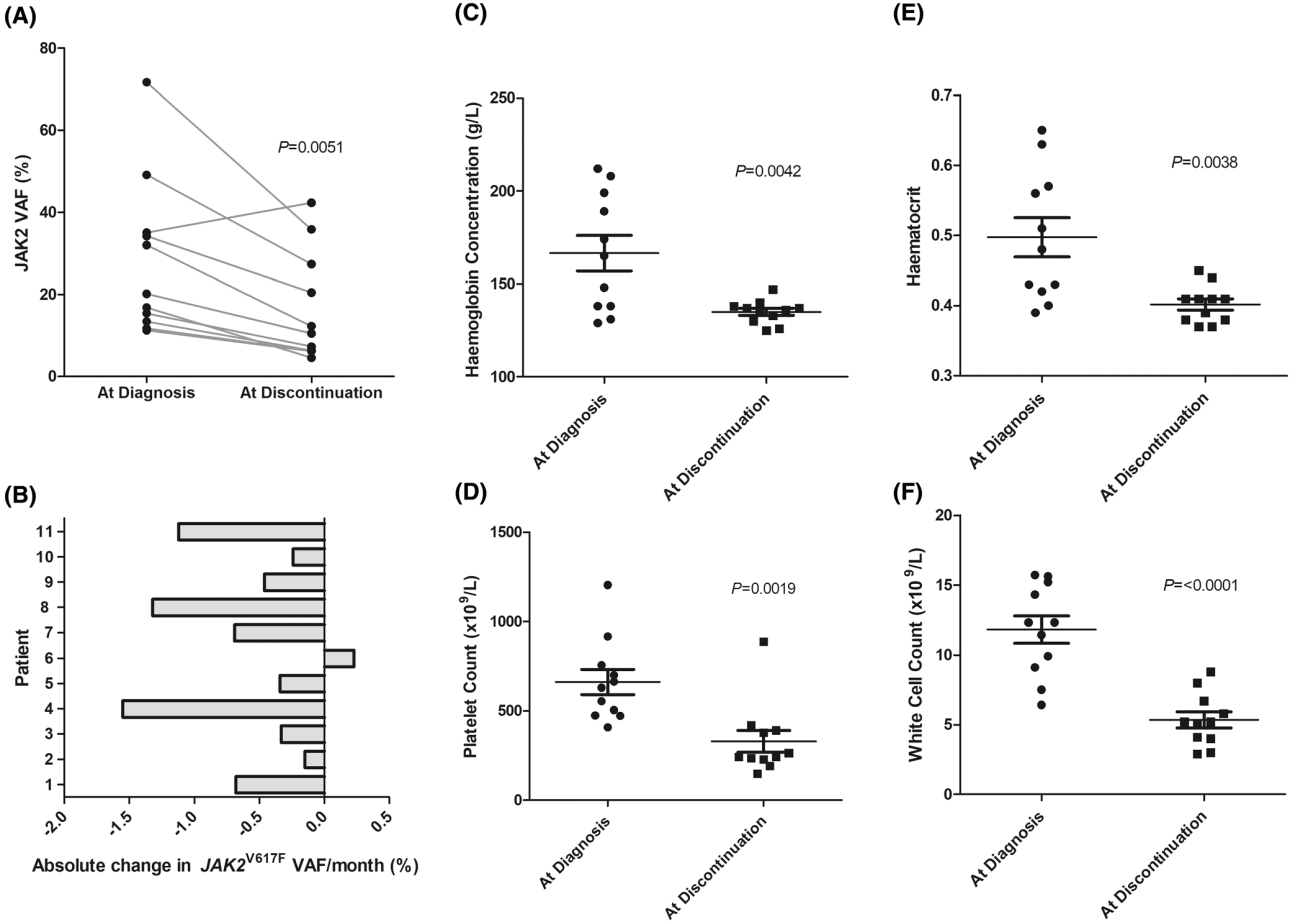
(A) Comparison of *JAK2* ^*V617F*^ variant allele frequency (VAF) between time of diagnosis and treatment discontinuation. *n* = 11 patients per group (B) Change in *JAK2* ^*V617F*^ VAF per month during treatment with PegIFNα. Change in haemoglobin concentration (C), platelet count (D), haematocrit (E) and white cell count (F) between time of diagnosis and treatment discontinuation. *n* = 11 patients per group.

Nine patients had a follow‐up sample collected 4–5 months after treatment discontinuation or at the time of treatment re‐initiation following haematological relapse, whichever was earlier. Of these, nine follow‐up patients, 55.6% (5/9), presented with frank haematological relapse associated with increased blood counts above the WHO 2022 diagnostic criteria within 6 months of treatment discontinuation. No clinical events, such as thromboembolic events, were observed in patients who had a haematological relapse off treatment. Patients who maintained TFR, defined as those that did not recommence treatment within the 6‐month follow‐up, had a significantly lower *JAK2* ^V617F^ VAF at the time of treatment discontinuation (Figure 2A). Previous data have suggested a cut‐off of 10% *JAK2* ^V617F^ VAF at the time of treatment discontinuation as a marker for improved TFR success.^10^ Our data corroborate this finding, with all patients failing to maintain TFR having a *JAK2* ^V617F^ VAF above 10% at the time of treatment discontinuation, while all those maintaining TFR at 6 months post‐discontinuation had a *JAK2* ^V617F^ VAF below 10% (Table S1). Interestingly, in our study, there was no significant difference in the change in *JAK2* ^V617F^ VAF following PegIFNα discontinuation between patients who went on to relapse within the first 6 months and those who maintained TFR (Figure 2B). This supports a previous study following seven MPN patients in which all patients maintained a complete haematological response despite four patients losing their molecular response.^17^ However, these studies are limited by their small patient cohort. It is important that increases in *JAK2* ^V617F^ VAF correlate with the risk of relapse if molecular monitoring is to be clinically beneficial. Therefore, confirming a correlation should be a priority in future trials.

**FIGURE 2:**
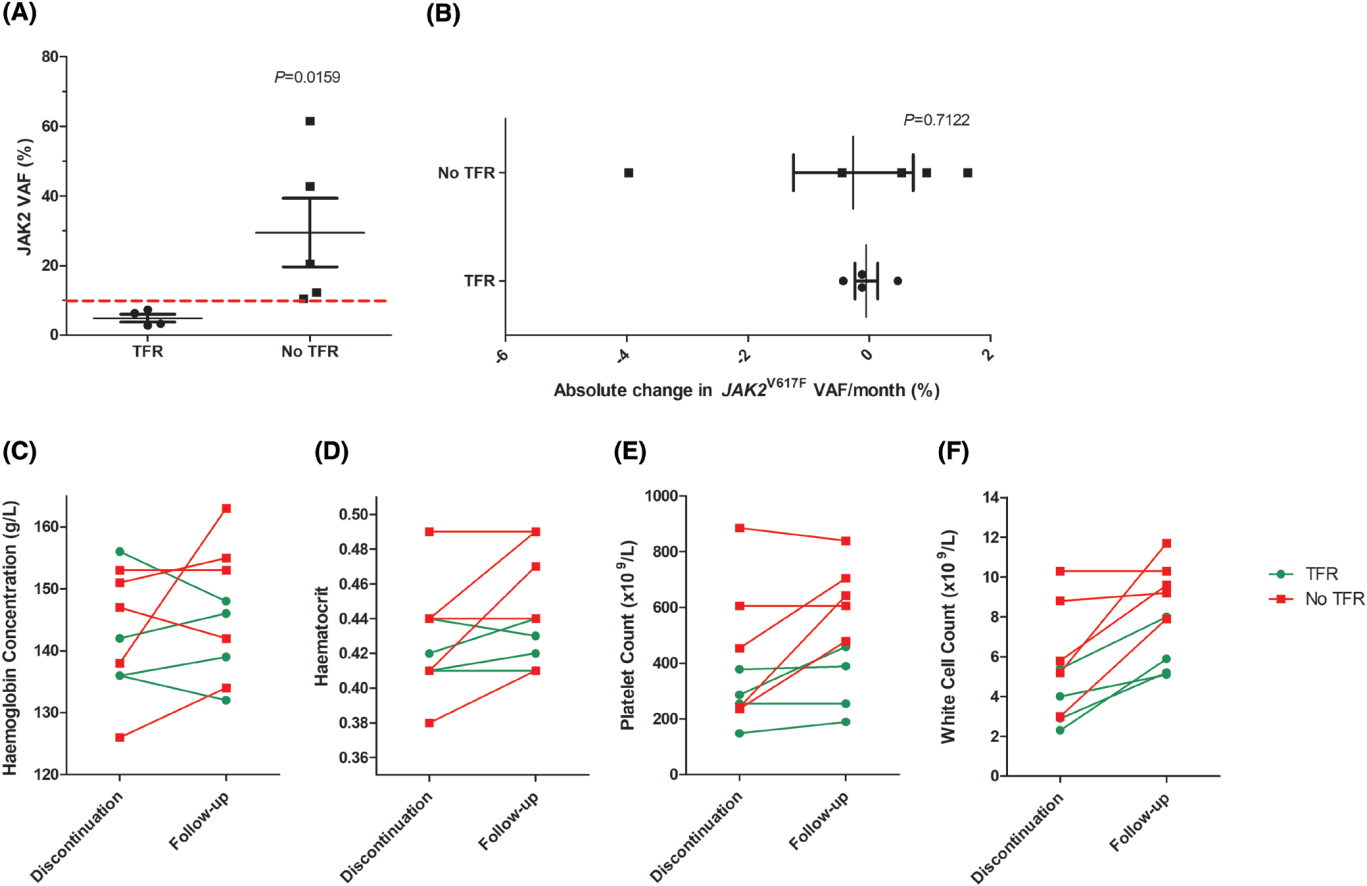
(A) Change in *JAK2^V617F^* VAF between patients who maintained or did not maintain treatment‐free remission (TFR) at 6 months post‐treatment discontinuation. Dotted line representing the 10% *JAK2* ^V617F^ VAF cut‐off. *n* = 4–5 patients per group (B) Change in *JAK2^V617F^* VAF between treatment discontinuation and follow‐up in patients who achieved TFR and those who did not achieve TFR. Change in haemoglobin concentration. *n* = 4–5 patients per group (C), haematocrit (D), platelet count (E) and white cell count (F) between time of PegIFNα discontinuation and follow‐up or haematological relapse. *n* = 9 patients per group.

In terms of clinical outcomes, significant increases in platelet count and white cell count were observed between treatment discontinuation and follow‐up (Figure 2E,F). However, no significant change in haemoglobin concentration or haematocrit was seen (Figure 2C,D). Lymphopenia and a higher ratio of neutrophils to lymphocytes (NLR) have been associated with poorer prognosis in MPN patients.^18^ We examined the absolute lymphocyte number and NLR in this cohort at the time of treatment discontinuation and follow‐up and found no significant differences between patients who maintained TFR and those who did not at either time point (Figure S3A,B). No significant difference in any of the other clinical outcomes was observed at the time of treatment discontinuation between patients who maintained TFR and those who did not (Figure 2C–F). This result may be impacted by the low patient numbers included in the follow‐up dataset. However, a previous study of ruxolitinib in MPN has also demonstrated a lack of correlation between normalisation of clinical outcomes including blood counts, symptoms and splenomegaly and molecular responses, indicating that blood counts alone may not fully represent the anti‐clonal activity of treatments such as ruxolitinib and PegIFNα.^16^

These preliminary data are in keeping with larger clinical trials demonstrating a haematological and molecular response in MPN patients treated with PegIFNα. Additionally, these data correlate with previously published data by De Olivieria et al. demonstrating that a *JAK2* ^V617F^ VAF of above 10% at the time of treatment discontinuation is independently associated with a higher cumulative incidence of relapse after IFN discontinuation.^10^ Interestingly, increases in *JAK2* ^V617F^ VAF following treatment discontinuation did not appear to correlate with increased risk of haematological relapse in this small cohort. The depth of remission achieved by patients in this study was notable, as no patients achieved an MMR despite long‐term treatment with PegIFNα. Previous data have shown that TFR can be maintained for up to 5 years in patients who have an MMR; however, this response may be less sustained in patients with partial molecular responses.^19^ The CONTINUATION‐PV trial showed an average *JAK2* ^V617F^ VAF of 5.6% and 17.9% in low‐ and high‐risk patients, respectively, after 6 years of treatment with BESREMi. Unlike CML, achieving long‐term MMR before treatment discontinuation may not be an appropriate target in MPN.^20^

This study is limited by the small cohort included and the short follow‐up time of patients' post‐treatment discontinuation. Further work will be needed to provide more rigorous data on TFR feasibility, optimal treatment duration, biomarkers of durable remission and achievable length of TFR. In particular, determining clinically relevant cut‐offs for the use of *JAK2* ^V617F^ as a target for molecular monitoring of MPN patients both on and off treatment will be important to provide clinical actionability. The role of molecular monitoring and potential benefits of disease modification in MPNs have been evaluated in a recent review by Claire Harrison.^21^ Ultimately, individualised therapy guided by molecular monitoring may facilitate a more targeted and less toxic approach to long‐term disease control in MPNs.

## AUTHOR CONTRIBUTIONS

Ryan Brown and Rebekah Bennett analysed the data. Ryan Brown wrote the paper. Clare Crean, Andrew Hindley, Julie McGimpsey, Nicholas Cunningham, Ciaren Graham, Graeme Greenfield, Mary Frances McMullin and Mark A. Catherwood provided critical analysis of the manuscript. Nicholas Cunningham, Aaron Niblock, Suzanne McPherson, Kathryn Boyd, Allister Foy and Mary Frances McMullin provided patient and clinical information. RBrown, MFM and MC designed the research study.

## FUNDING INFORMATION

This study was funded by the regional molecular diagnostic service as part of a service improvement project.

## CONFLICT OF INTEREST STATEMENT

The authors declare no competing interests.

## CONSENT

Patient consent was attained for the collection of PB samples used in this study. All PB samples were collected as part of routine clinical monitoring.

## Supporting information


Figures S1–S3.

Table S1.

## Data Availability

The data that support the findings of this study are available from the corresponding author upon reasonable request.
