# Supplementary material for: The impact of JAK2 V617F variant allele frequency in MPN patients following PEGylated interferon alpha discontinuation
Source: Br J Haematol. 2025 Oct 24;207(6):2597–601. doi: 10.1111/bjh.70198 (PMC12710198; doi:10.1111/bjh.70198)
Supplement: Supplementary file 1 — Figures S1–S3. [file BJH-207-2597-s001.docx]

**Supplementary methods**

Study cohort

A cohort of 15 patients diagnosed with an MPN according to the WHO 2022 classification criteria and who discontinued treatment with Pegylated IFN alpha (PegIFNα) between September and December 2024 were included in this study. A decision to discontinue PegIFNα treatment due to a sudden interruption in Pegasys supply rather than switch to an alternative therapy was based on whether the patient’s blood counts including haemoglobin concentration, haematocrit and platelet count were in the normal range and was independent of time on treatment. In each case the decision to discontinue treatment was made by the clinical team in concurrence with the patient. Patients were made aware of the potential risks of treatment discontinuation including the potential risk of thromboembolic events and instructed to inform the clinical team immediately of any symptoms developed while off treatment. Additionally, patient blood counts were monitored at review clinics every 8-12 weeks to ensure blood counts remained within the normal range thereby limiting the risk of thromboembolic events. A peripheral blood (PB) sample was collected at the point of treatment discontinuation and at a follow-up review four to five months post discontinuation. Following haematological relapse, as indicated by the return of MPN-typical changes in the PB (erythrocytosis/thrombocytosis) or clinical symptoms, treatment was reinitiated and an additional PB sample collected.

Quantitative PCR

Quantitative PCR (qPCR) was performed on DNA extracted from peripheral blood using a Roche LightCycler 480 PCR system with custom PCR primers for *JAK2* WT reverse (GTAGTTTTACTTACTCTCGTCTCCACATAC) (Eurofins), *JAK2*^V617F^ reverse (GTAGTTTTACTTACTCTCGTCTCCACATAA) (Eurogentec), common *JAK2* forward (CTTTCTTTGAAGCAGCAAGTATGA) (Eurofins), and custom JAK2V617F probe (6FAM-TGAGCAAGCTTTCTCACAAGCATTTGGTTT-TAMRA) (ThermoFisher). WT and *JAK2*^V617F^ copy number were determined by comparison to a standard curve. Standard curves for WT and *JAK2*^V617F^ were generated from the WHO 1st International Reference Panel for Genomic *JAK2*^V617F^ 16/120. VAF was determined by calculating the percentage of *JAK2*^V617F^ mutant copy number to total *JAK2*^V617F^ copy number. This qPCR assay has been previously described (22).

Statistics

All data were analysed using GraphPad PRISM and are reported as mean ± SEM. Statistical analyses were performed using Student’s t test and P < 0.05 was accepted to indicate statistical significance.

**Supplementary Figures**

**Figure S1.** Choice of treatment following haematological relapse and treatment re-initiation.

**
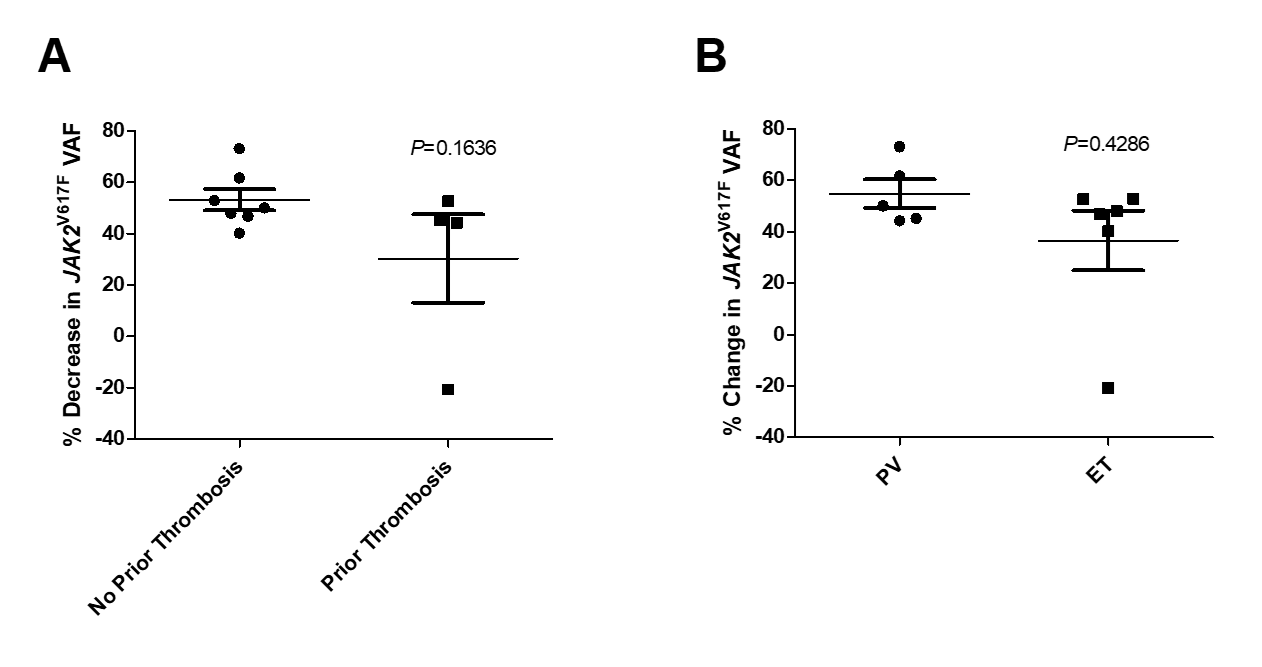
**

**Figure S2.** Percentage decrease in *JAK2*^V617F^ VAF between treatment initiation and discontinuation in (A) patients with or without prior thrombotic events, n = 4-7 patients per group and (B) patients with PV or ET n = 5-6 patients per group.

**Figure S3.** (A) Absolute lymphocyte counts and (B) neutrophil to lymphocyte ratio (NLR) at the time of treatment discontinuation and follow-up in patients that maintained treatment free remission (TFR) and patients that did not maintain TFR. n= 4-5 patients per group.

**Supplementary Table 1.** Patient demographics for MPN cohort included in the study. (NA = not available).

**References**

16. Guglielmelli P, Mora B, Gesullo F, Mannelli F, Loscocco GG, Signori L, *et al.* Clinical impact of mutated JAK2 allele burden reduction in polycythemia vera and essential thrombocythemia. *American Journal of Hematology* 2024;99:1550–1559.

17. Larsen TS, Møller MB, De Stricker K, Nørgaard P, Samuelsson J, Marcher C, *et al.* Minimal residual disease and normalization of the bone marrow after long-term treatment with alpha-interferon2b in polycythemia vera. A report on molecular response patterns in seven patients in sustained complete hematological remission. *Hematology* 2009;14:331–334.

18. Larsen MK, Skov V, Kjær L, Eickhardt-Dalbøge CS, Knudsen TA, Kristiansen MH, *et al.* Neutrophil-to-lymphocyte ratio and all-cause mortality with and without myeloproliferative neoplasms—a Danish longitudinal study. *Blood Cancer J* 2024;14:1–12.

19. Utke Rank C, Weis Bjerrum O, Larsen TS, Kjær L, De Stricker K, Riley CH, *et al.* Minimal residual disease after long-term interferon-alpha2 treatment: a report on hematological, molecular and histomorphological response patterns in 10 patients with essential thrombocythemia and polycythemia vera. *Leuk Lymphoma* 2016;57:348–354.

20. Kiladjian JJ, Klade C, Georgiev P, Krochmalczyk D, Gercheva-Kyuchukova L, Egyed M, *et al.* Efficacy and Safety of Long-Term Ropeginterferon Alfa-2b Treatment in Patients with Low-Risk and High-Risk Polycythemia Vera (PV). *Blood* 2022;140:9663–9664.

21. Harrison CN. Are we ready for disease modification in myeloproliferative neoplasms? *HemaSphere* 2024;8:e70003.

22. Jovanovic, J. v., Ivey, A., Vannucchi, A. M., Lippert, E., Oppliger Leibundgut, E., Cassinat, B., Pallisgaard, N., Maroc, N., Hermouet, S., Nickless, G., Guglielmelli, P., van der Reijden, B. A., Jansen, J. H., Alpermann, T., Schnittger, S., Bench, A., Tobal, K., Wilkins, B., Cuthill, K., … Grimwade, D. (2013). Establishing optimal quantitative-polymerase chain reaction assays for routine diagnosis and tracking of minimal residual disease in JAK2-V617F-associated myeloproliferative neoplasms: A joint European LeukemiaNet/MPN&MPNr-EuroNet (COST action BM0902) study. Leukemia, 27(10), 2032–2039. https://doi.org/10.1038/LEU.2013.219,
