# Supplementary material for: The impact of JAK2 V617F variant allele frequency in MPN patients following PEGylated interferon alpha discontinuation
Source: Br J Haematol. 2025 Oct 24;207(6):2597–601. doi: 10.1111/bjh.70198 (PMC12710198; doi:10.1111/bjh.70198)
Supplement: Supplementary file 2 — Table S1. [file BJH-207-2597-s002.docx]

| **Patient** | **Age** | **Gender** | **Diagnosis** | **Thrombosis History** | **Time on PegIFNα (Months)** | **JAK2 VAF at Diagnosis** | ***JAK2^V617F^* VAF at Discontinuation** | **Time to follow-up (Months)** | ***JAK2^V617F^* VAF at Follow-up** | **Recommenced Treatment at follow-up** |
| --- | --- | --- | --- | --- | --- | --- | --- | --- | --- | --- |
| 1 | 76 | F | PV | No | 18 | 16.79% | 4.52% | N/A | N/A | N/A |
| 2 | 63 | F | ET | No | 46 | 13.39% | 6.31% | N/A | N/A | N/A |
| 3 | 63 | M | ET | No | 42 | 34.20% | 20.44% | 3 | 19.10% | Yes |
| 4 | 57 | F | PV | Yes | 14 | 49.11% | 27.35% | N/A | N/A | N/A |
| 5 | 57 | F | PV | Yes | 3 | 11.20% | 6.14% | N/A | N/A | N/A |
| 6 | 70 | F | ET | Yes | 31 | 35.00% | 42.27% | N/A | N/A | N/A |
| 7 | 73 | M | ET | No | 14 | 20.10% | 10.47% | 1 | 12.00% | Yes |
| 8 | 49 | M | PV | No | 13 | 31.97% | 12.24% | 4 | 16.01% | Yes |
| 9 | 64 | F | ET | No | 12 | 11.72% | 6.23% | 5 | 4.10% | No |
| 10 | 56 | F | ET | Yes | 30 | 15% | 7.27% | 4 | 9.14% | No |
| 11 | 70 | M | PV | No | 30 | 71.67% | 35.82% | N/A | N/A | N/A |
| 12 | 52 | M | PV | No | 87 | NA | 61.51% | 1 | 57.54% | Yes |
| 13 | 56 | F | PV | No | 94 | NA | 42.72% | 5 | 45.35% | Yes |
| 14 | 49 | M | PV | No | 84 | NA | 3.23% | 5 | 2.62% | No |
| 15 | 59 | M | ET | No | 107 | NA | 2.74% | 5 | 2.15% | No |
